# Joint hypermobility syndrome in children with idiopathic scoliosis

**DOI:** 10.1186/1748-7161-7-S1-O69

**Published:** 2012-01-27

**Authors:** D Czaprowski, T Kotwicki, P Pawlowska, L Stolinski

**Affiliations:** 1 Józef Rusiecki University College in Olsztyn, Poland; 2 University of Medical Sciences, Poznań, Poland; 3 Rehasport Clinic; Sports Secondary School Complex the John Paul IIPoznań; Skierniewice, Poland

## Purpose of study

To assess the frequency of occurrence of the hypermobility syndrome (HS) in children and teenagers with idiopathic scoliosis (IS). To assess the presence of HS in relation to the angle of curvature, vertebral rotation, length of scoliosis and the treatment used.

## Background

Joint hypermobility syndrome is diagnosed when the mobility of small and large joints is increased in relation to standard mobility for any given age, gender and race, and after excluding systemic diseases [1][2][3]. It is assessed by clinical examination using specific scales (Beighton) [4]. Some methods of physiotherapy used to treat scoliotic children, include exercises that aim at increasing the range of spinal mobility to achieve curve correction [5][6][7][8][9].

## Materials and methods

128 children (92 girls and 36 boys) aged 9 to 18 years, comprising 70 IS children (34 single and 36 double IS), Cobb angle from 11 to 53 degrees, and 58 scoliosis-free controls were examined. Beighton scale as well as Hakim and Grahame questionnaire were used to disclose the presence of HS [1][4][9].

## Results

HS was noted more often in children with scoliosis than in the control group (p<0.0001). The angle of curvature, the apical vertebra rotation, the number of vertebrae of the primary curve and the treatment (brace or physiotherapy) did not influence the frequency of occurrence of HS. In single curve IS, the HS appeared more often than in double curve IS (p=0.03).

## Conclusions

HS appears more often in children with IS than in healthy controls, especially in single curves. There was no relation of HS with the angle, rotation, length of scoliosis or treatment type. HS should be taken into account when physiotherapy is planned in IS children.

## References

[B1] Hakim A Grahame R Joint hypermobility Best Practice & Research Clinical Rheumatology 2003 17 6 989 1004 10.1016/j.berh.2003.08.001 15123047

[B2] De Inocencio AJ Casas OI Ortiz BL Joint hypermobility: prevalence and relationship with musculoskeletal pain Anales de Pediatria (Barc) 2004 61 162 66 10.1157/13064596 15274882

[B3] Adib N Davies K Grahame R Woo P Murray KJ Joint hypermobility syndrome in childhood. A not so bening multisystem disorder? Rehumatol 2005 44 744 50 10.1093/rheumatology/keh557 15728418

[B4] Beighton P Grahame R Bird H Hypermobility of joints 1999 3 London:Springer

[B5] Bialek M M'Hango A Grivas TB FITS Concept Functional Individual Therapy of Scoliosis. In The Conservative Scoliosis Treatment. 2008 Amsterdam: IOS Press 250 261 18401096

[B6] Karski T Kalakucki J Karski J “Syndrome of Contractures” (Acording to Mau) with the Abduction Contracture of the Right Hip as Causative Factor for Development of the So-Called Idiopathic Scoliosis. Studies in Health Technology and Informatics 2006 123 34 39 17108400

[B7] Bauknecht K Scoliosis dance therapy: a worth-while addition to conservative scoliosis treatments? A pilot study evaluating the effect of a DVD led instruction on the wellbeing of scoliosis sufferers. 8th Annual Meeting of the SOSORT, International Conference on Conservative Management of Spinal Deformities, Barcelona 2011

[B8] Sastre S Lapuente JP Santapau C Bueno M Dynamic Treatment of Scoliosis (The Results of 174 Cases). Research into Spinal Deformities 1999 Amsterdam:IOS Press

[B9] Hakim AJ Grahame R A simple questionnaire to detect hypermobility: an adjunct to the assessment of patients with diffuse musculoskeletal pain. International Journal of Clinical Practice 2003 57 163 166 12723715

